# The Bonny Method of Guided Imagery and Music as a trauma-informed approach in eating disorder treatment: theoretical framework and case illustrations

**DOI:** 10.1186/s40337-026-01625-7

**Published:** 2026-04-30

**Authors:** Annie Heiderscheit

**Affiliations:** Cambridge Institute for Music Therapy Research, Anglia Ruskin University, Cambridge, UK

**Keywords:** Eating disorders, Music psychotherapy, Bonny Method of Guided Imagery and Music, Trauma-informed care, Emotion regulation

## Abstract

**Background:**

Eating disorders (EDs) are severe and complex mental health conditions with high mortality, significant psychiatric comorbidity, and increasing global prevalence. ED behaviours (e.g., restriction, bingeing, purging) may function as adaptive strategies for affect regulation, management of trauma-related distress, and avoidance of embodied experience. While trauma-informed care emphasises safety, empowerment, and relational attunement, trauma in EDs is often held somatically and may remain inaccessible through verbal therapies alone. The Bonny Method of Guided Imagery and Music (GIM), an experiential depth-oriented music psychotherapy, uses therapist-selected programmed music to evoke imagery, emotion, memory, and embodied processes within a supportive therapeutic relationship. This paper explores GIM as a trauma-informed approach targeting psychological and embodied mechanisms underlying ED symptoms. A theoretical framework is outlined in which music-evoked imagery and neuroaesthetics engagement facilitate symbolic emotional exploration, embodied integration, interoceptive reconnection, affect regulation within safety, and meaning-making and identity repair.

**Case presentation:**

The framework is illustrated through four case examples from a qualitative feasibility study of GIM in ED treatment. Participants were four adult women (aged 26–57) engaged across levels of care, each receiving 12–16 individual GIM sessions over 12-months. The cases include selected session excerpts highlighting music and imagery processes that explore the functional role of ED symptoms.

**Conclusions:**

Across cases, GIM enabled access to unresolved emotions, trauma-related somatic distress, attachment dynamics, and identity disturbances, while supporting emotion regulation, agency, and transformation of symptom-related coping. These findings position GIM as a promising multimodal, embodied intervention for trauma-informed ED treatment, particularly for individuals with complex trauma and difficulties engaging in verbal interventions. Further research is needed to clarify mechanisms of change, clinical indications, and integration within multidisciplinary ED care.

## Introduction

Eating disorders (EDs) are complex mental health conditions characterised by eating-related behaviours that negatively impact nutritional intake, body image, and weight, often resulting in medical complications and affecting psychological and psychosocial wellbeing [1, 2]. ED diagnoses include anorexia nervosa (AN), bulimia nervosa (BN), binge eating disorder (BED), and other specified feeding or eating disorders (OSFED) and typically have an onset during adolescence, with an average duration of six years, persisting into adulthood [3–5]. Further, individuals living with an ED have a higher mortality rate, which is six times higher for those with AN and double for other EDs [1, 6, 7].

The aetiology of EDs is widely understood as multifactorial and often conceptualised through a biopsychosocial framework in which biological, psychological and sociocultural factors interact dynamically to influence risk, onset, and maintenance [8]. Biological contributors include genetic vulnerability, neurobiological alterations, and dysregulation in appetite and reward systems [9]. Psychological factors include perfectionism, low self-esteem, and emotion dysregulation [10], while social and environmental influences include cultural body ideals, interpersonal relationships, exposure to weight-related stigma, which shape the development and persistence of disordered eating behaviours [11]. These factors do not operate independently but interactive in complex and reciprocal ways, contributing to illness presentation and trajectories [12].

Incidences of EDs have increased significantly over the past two decades, with a global burden report indicating a 40–50% increase [12]. In 2019, it was estimated that globally over 55 million people were living with an ED and during the COVID-19 pandemic there was a 15% rise in the incidence of EDs, as well as symptom severity, deterioration and comorbidities [13–18]. As a result, individuals living with an ED were more likely to present with a complex profile due to severity of symptoms and the prevalence of mental health comorbidities [18].

Most individuals living with an ED meet criteria for at least one additional mental health condition [19]. Mood disorders, especially major depression are most prevalent, affecting nearly 50% of ED patients and contributing to illness severity and suicide risk [20–23]. Anxiety disorders (generalised anxiety, social anxiety and OCD) are also widespread, and evident in between 30 and 50% of clinical samples, with obsessive–compulsive symptoms associated with restrictive behaviors [20]. Trauma-related symptoms and PTSD occur at elevated rates in individuals with BN and BED, where interpersonal trauma is more common [17, 18]. Substance use disorders (SUDs) disproportionately occur in BN and BED, where shared traits of impulsivity and affect-driven dysregulation are represented [20, 21]. Personality disorders (borderline and avoidant) are evident in 30–50% of ED patients and are often associated with poorer outcomes [24]. Further, EDs can share behaviour patterns of internalising conditions (inner emotional struggles) and externalising conditions (impulsive or risk-taking behaviours), indicating shared underlying vulnerabilities with mental health conditions [25–27].

## Psychological processes underlying eating disorder behaviours

ED behaviours are often understood as functional responses to psychological distress rather than maladaptive responses to food and weight [28]. Through this lens restrictive eating patterns, bingeing, purging, and compulsive exercise can serve as strategies to help regulate emotions, manage trauma-related symptoms, and foster a sense of safety to protect oneself [29]. Individuals with EDs frequently describe their symptoms as a way of providing relief from difficult emotions, fostering a sense of control in contexts of chaos, or numbing their internal experience of shame, fear, and relational pain [30, 31].

**Fig. 1:**
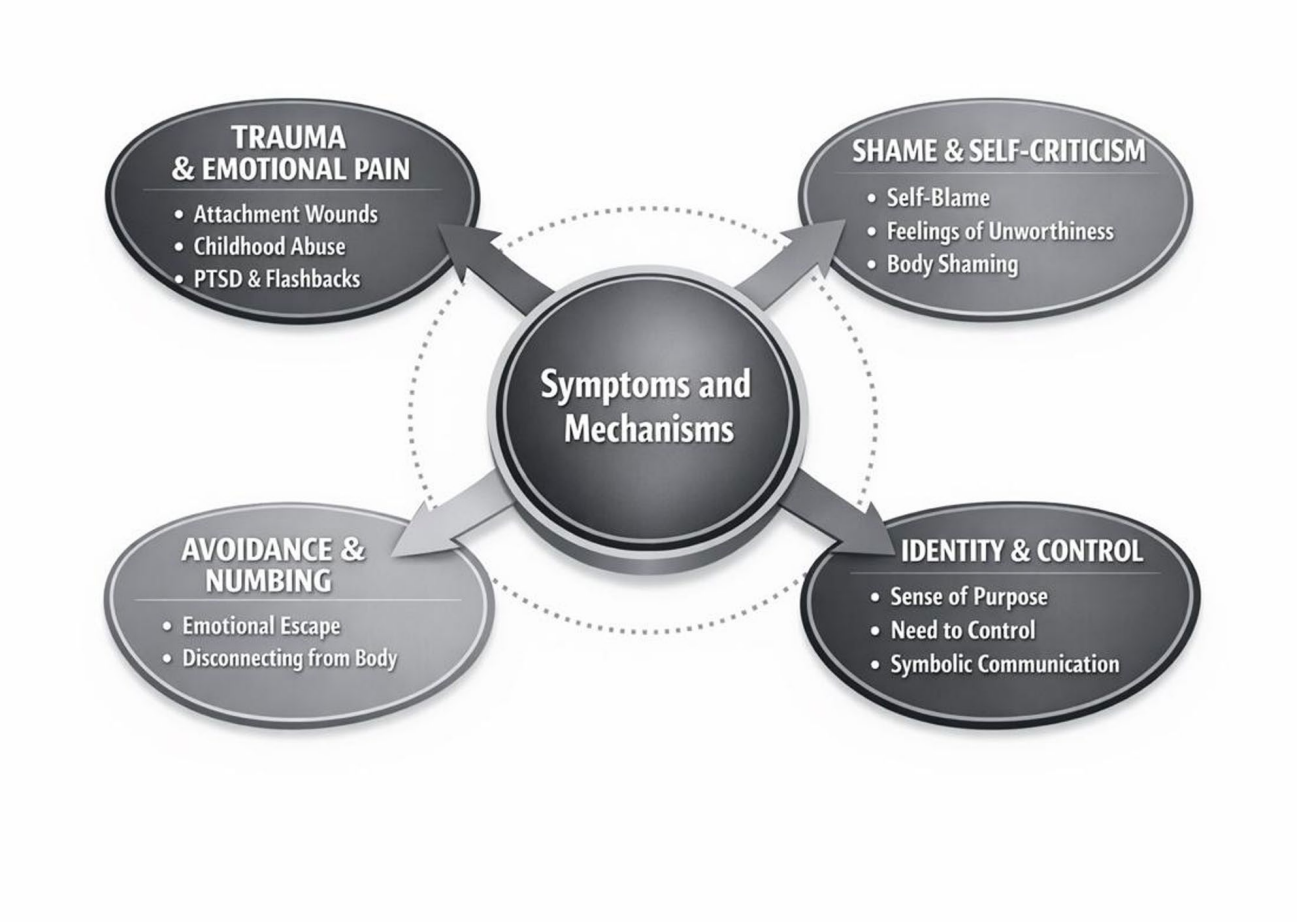
ED symptom causes and mechanisms

Figure 1 illustrates a perspective of the psychological processes underlying ED behaviours. Research indicates people with an ED have elevated exposure to interpersonal trauma (childhood abuse, neglect, and attachment disruption) compared to the general population [30, 31]. Post-traumatic stress symptoms including intrusive memories, avoidance, and fear of persistent threat interfere with emotional processing. As a result, ED behaviours may function as attempts to modulate trauma-related hyperarousal or hypo arousal states to provide temporary regulation [32]. ED behaviours of restriction and ritualised eating may be used to reduce perceived vulnerability by narrowing attentional focus and dampening affect, whereas bingeing and purging may be utilized to interrupt dissociative or emotionally overwhelming states [33]. These findings align with trauma-informed practice that understand ED symptoms as adaptations to threat, rather than pathology [27].

**Fig. 2:**
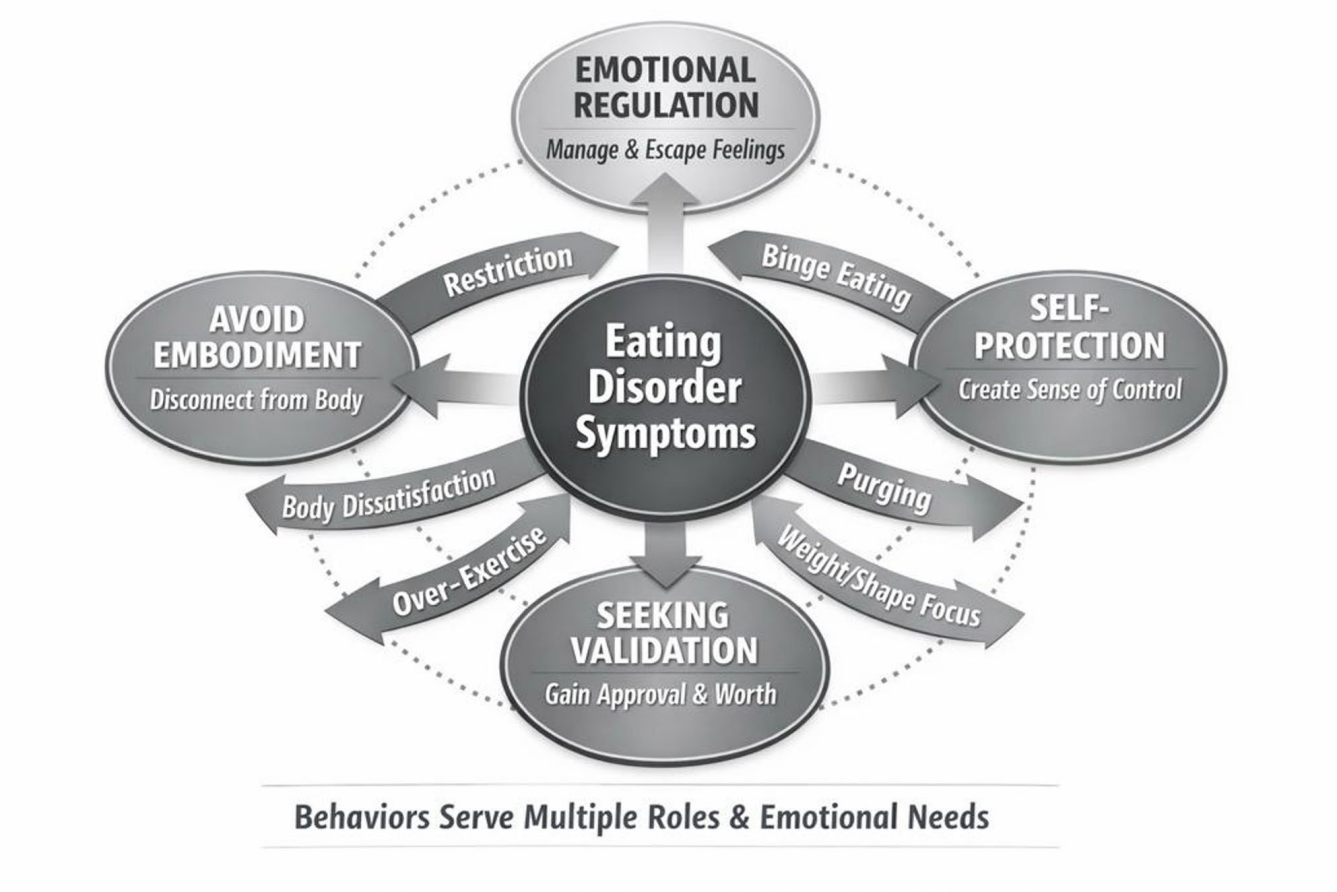
Function and interconnectedness of eating disorder symptoms/behaviours

This is represented in how shame and harsh self-criticism are affective drivers of ED maintenance, particularly with restrictive behaviors [29]. Individuals with EDs internalise blame and experience their body as an illustration of failure, leading to compulsive behaviours of food and weight control to self-correct [29]. ED behaviours also serve identity-regulating functions, offering structure, purpose, or a coherent sense of self when other aspects of identity feel unsafe or unavailable [25, 29], [34]. As a result, symptoms become intertwined with self-concept, which can result in making changes feel threatening to psychological survival [34, 34–36]. Figure 2 illustrates the different functions that ED symptoms and behaviours serve and their interconnected nature. Individuals with EDs have described eating, fullness, and hunger as emotionally dangerous experiences that trigger memories, affective states, or relational meanings, leading to restriction and purging to avoid embodiment and minimize contact with internal sensations [33–35]. This avoidance can reinforce body image disturbances and perpetuate reliance on the ED behaviours as a primary means of coping [37, 38].

Research increasingly conceptualises EDs as a complex, dynamic network of interrelated symptoms that interact, and that only contribute to the onset, sustaining maintenance, and evolution of ED symptoms and behaviours over time [39]. EDs engage cognitive, affective, and behavioural components that influence each other and operate through shared mechanisms (emotion dysregulation, self-criticism, and interpersonal processes) [10, 40]. Emerging research suggests that ED symptoms intersect with other psychological symptoms and neurodevelopmental features, that indicates a pattern of how ED symptoms co-occur, influence and reinforce one another through shared cognitive and emotional pathways [41]. This interconnected nature of ED and psychological symptoms helps explain why behaviours persist, shift, or expand into new patterns over time [42]. This also explicates why interventions that address underlying mechanisms that improve emotion regulation, reduce avoidance, and transform maladaptive beliefs about the body are warranted, rather than focus on the interruption of surface behaviours [42–46].

Emerging research also suggests that ED symptoms may act as symbolic representation and communication of unmet relational needs, particularly when expression of distress feels unsafe or ineffective [38]. For individuals with families impacted by trauma or emotional invalidation, symptoms may serve an unanswered cry for help or a means of making suffering visible when words fail [29, 31]. This understanding of ED behaviours helps explain the persistence of symptoms despite their negative consequences [27]. Further, it highlights the importance of trauma-informed and relationally-attuned treatment approaches [44–46].

### The Bonny Method of Guided Imagery and Music

The Bonny Method of Guided Imagery and Music (GIM) is a music psychotherapy approach utilising focused listening to therapist selected, programmed music in a relaxed state [47]. The music functions as a catalyst and container, eliciting feelings, images, thoughts, and sensations, throughout the experiential process [48]. The therapist’s verbal prompting and support helps to evoke imagery, facilitate exploration, and foster emotional processing, enabling the client to access material, explore affective states, and engage in making meaning within a safe therapeutic space [48, 49].

Within each GIM session, the music is selected to address the unique needs of the client, with a focus on meeting them where they are in their therapeutic process, and can include transitioning the music to support emerging and changing needs [49, 50]. Sessions begin with a check in to identify and share developments or insights from the week or previous session [42]. This information is vital to informing the therapist’s clinical decision making in selecting the music and how to best support the client’s transition to a relaxed state and into the music and imagery experience [50]. While the client listens to the music and they are actively imaging and verbally reporting what they are experiencing. The therapist verbally responds to support the client’s exploration of images, feelings, sensations, and memories [47, 50].

The music in GIM is carefully structured and sequenced, traditionally utilising Western classical music because of its dynamic range, structural depth, and emotional expressiveness, modern adaptations may incorporate other genres [48, 50]. The music is typically instrumental rather than lyrical; this is intentional as the absence of words reduces cognitive distraction and allows the listener to project their own meanings and imagery onto the music, facilitating a more personal and symbolic experienced [50]. When music with lyrical content is integrated it is in a language that the listener is not fluent in. In GIM, the music is not an arbitrary background sound; it is a primary therapeutic agent that is carefully chosen and programmed to shape the client’s inner imagery experience, emotional flow, and depth of exploration [50]. Music programs range in length from 30 to 60 min and as they listen, imagery unfolds. Their focus may move from one area or aspect of their life to another [51]. In this process, GIM is like a scanner, it surveys the client’s whole universe, identifies priorities for exploration, and then leads the client into experiences that either focus directly on the most pressing issue or provides a context where the issue can be explored at various distances and from different perspectives [45].

In this way, GIM affords the client access to primary and underlying issues, aimed at addressing core therapeutic needs and issues [49, 52]. As a result, the imagery that emerges, as well as their engagement and responses, are key to supporting change and therapeutic growth [49, 50]. When the music concludes, the therapist guides the client to bring their imagery to a close and focus on the physical space around them. Following the music and imagery experience, the therapist facilitates a reflection and discussion of the experience to foster integrating insights and meaning [52]. This therapeutic work is often completed within a series of GIM sessions that is dependent on the depth or complexity of the therapeutic issues [53, 54].

## GIM research in mental health and eating disorder treatment

GIM has been implemented across diverse clinical populations, including in mental health and substance-use disorder (SUD) treatment [54–59]. Qualitative research indicates that GIM helped clients regulate their emotions and gain insight into their self-defeating or harmful behaviours [57]. GIM sessions during inpatient SUD treatment from a randomized controlled trial, indicated that sessions helped clients uncover and address unresolved issues of grief and loss, access and express repressed emotions, resolve intrapersonal conflicts, and work through feelings and experiences related abandonment and abuse [54, 55]. Qualitative and GIM case study research with individuals with a trauma history or PTSD indicates improved affect regulation, decrease in dissociative episodes, and containment of emotions and improved capacity to process emotions related to the trauma [58–61]. This research provides support for the exploration of the use of GIM in ED treatment, due to similar challenges in emotional regulation, disrupted or impaired interoception, and high incidence of trauma [50, 53].

A feasibility study examining the use of GIM with adults in ED treatment found that GIM supported the exploration of unresolved emotions, intrapersonal conflict and trauma [50]. Participants described the music and imagery process as helping to access emotions that were difficult to experience and express, which helped in developing insights, self-compassion, and making changes in relational patterns [50]. This embodied approach afforded clients opportunities to discover inner resources and gain confidence in their abilities, fostering their sense of agency and empowerment [62]. Related qualitative research with Music Breathing, a structured, GIM-derived method, demonstrates potential for supporting emotion regulation capacities for individuals engaged in ED treatment [63]. These findings highlight the promise of GIM as a therapeutic approach to address the affective, cognitive, and embodied dimensions of ED behaviours and the complex needs of those in ED treatment.

Despite these contributions, the GIM literature in ED treatment remains limited, and conceptual development is needed to articulate how the method can be systematically applied to explore and work through the unresolved issues that function to support ED symptoms. Given the significance of emotional dysregulation, identity disturbance, and disrupted embodiment in EDs [64, 65], GIM offers a unique multimodal pathway to foster therapeutic engagement, integrating music, imagery, creative and symbolic processes, within the support of a therapeutic relationship. This paper explores these emerging areas by examining the use of GIM as a clinical approach for uncovering and addressing the root causes of ED symptoms. This is explicated through the presentation of four case illustrations of adult clients engaged in ED treatment.

## Theoretical framework

**Fig. 3:**
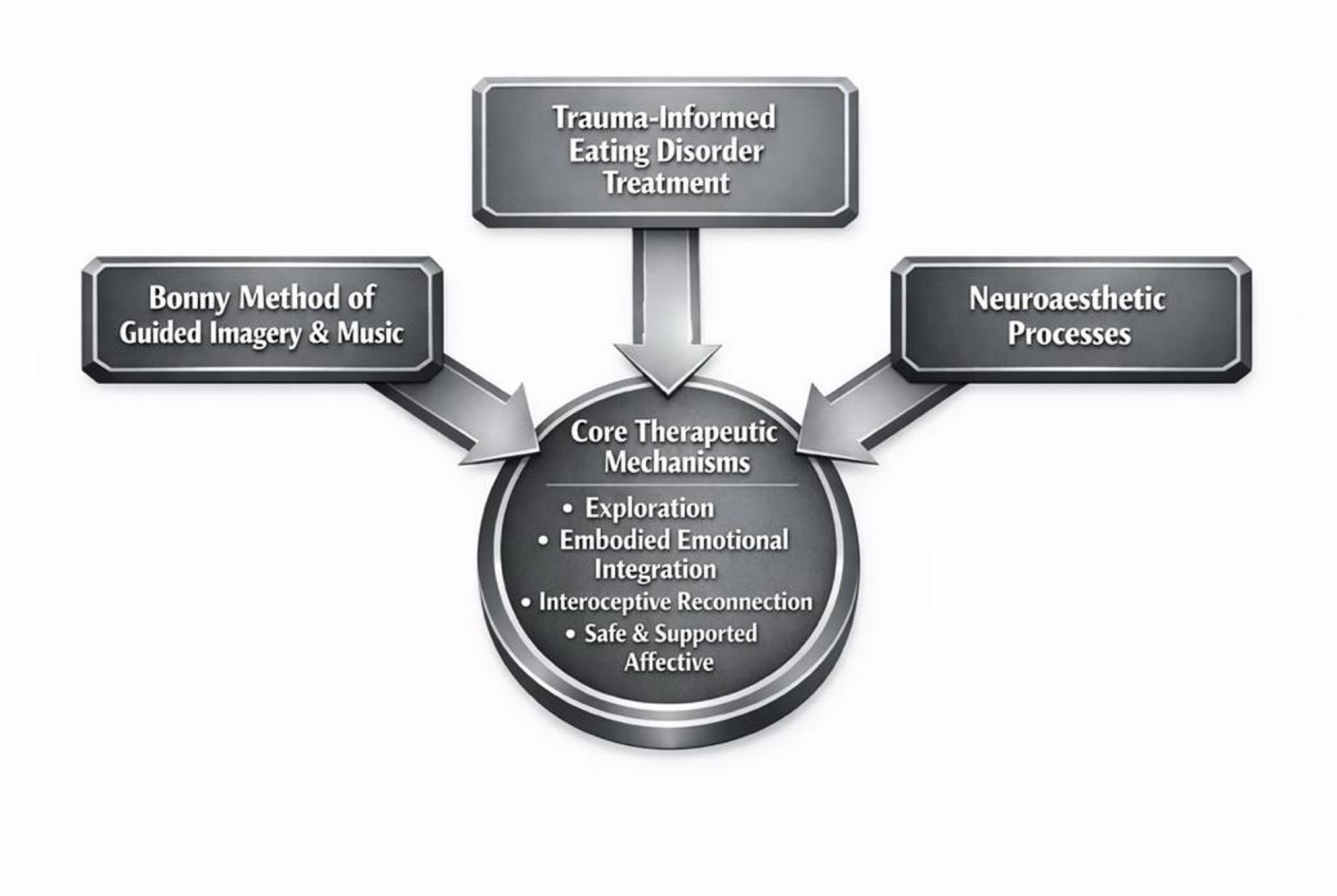
Theoretical framework

To conceptualise the clinical relevance of GIM within trauma-informed ED treatment, Fig. 3 provides a theoretical framework indicating how the music-evoked imagery process in GIM accesses and activates core mechanisms of recovery. Trauma-informed approaches emphasise safety, empowerment, and sensitivity to the nervous system impacts of trauma [66], yet many individuals with EDs experience distress and traumatic material as embodied, implicit, and difficult to access through verbal therapies alone [67]. Given the high prevalence of affect dysregulation, dissociation, and disrupted interoception in ED populations, experiential modalities that engage emotion and embodied awareness are increasingly relevant [61, 62].

Central to this framework is GIM, utilising music-evoked imagery within a structured therapeutic relationship. Music elicits imagery, emotion, memory, and bodily sensation while the therapist provides guidance that supports exploration and regulation [68, 69]. Neuroaesthetics research suggests that aesthetic engagement with music activates neural systems involved in affect, reward, memory, and self-referential processing, including limbic and interoceptive networks often disrupted in trauma and EDs [70–73]. As a result, music-evoked imagery provides a pathway for accessing emotional and somatic material in a psychologically safe and tolerable way.

Figure 3 illustrates the interaction between trauma-informed approach, music-evoked imagery (GIM), and neuroaesthetics engagement supporting several core therapeutic mechanisms: symbolic emotional exploration, embodied emotional integration, interoceptive reconnection, affect regulation within safety, and meaning-making and identity repair [74–76]. Symbolic imagery allows implicit emotional material to emerge in metaphorical form, while embodied listening supports reintegration of affect and embodied awareness often avoided through ED symptoms [73]. By fostering interoceptive tolerance and emotional containment, GIM helps to expand clients’ capacity to regulate distress without reliance on ED behaviours, while also supporting narrative coherence and recovery-oriented identity reconstruction [54–56] This framework provides the foundation for the case illustrations, demonstrating how these mechanisms emerge clinically in GIM sessions with adults in ED treatment.

## Methods

The case illustrations originated from a descriptive feasibility study that examined the implementation of GIM in ED treatment. Previous analyses of the study data examined clients’ perceived benefits and challenges [50] of GIM in ED treatment and thematic analysis of imagery content, identifying therapeutic themes and discoveries [54]. The data provided insight into what therapeutic issues clients reported they felt they were able to address in the GIM sessions, as well as therapeutic content that emerged during the music and imagery sessions.

The aim of this study was to theoretically articulate and explore the role of the Bonny Method of Guided Imagery and Music (GIM) as a creative aesthetic embodied, trauma-informed therapeutic approach for individuals with EDs, specifically where symptoms are rooted in emotional dysregulation and trauma-related affective. The case illustrations seek to address the growing recognition that ED behaviours function as adaptive coping strategies used to manage overwhelming emotions, traumatic distress, dissociation, and disrupted body experience. Further, because trauma in ED populations is experienced implicitly and somatically, these case illustrations highlight the limitations of interventions that utilise verbal processing exclusively.

### Rationale for case selection and analytic focus

While prior analyses of the full data set employed thematic and intertextual methods to identify recurrent imagery patterns and shared therapeutic themes, these approaches are abstracted across cases, privileging cross-case convergence over case depth [50, 54]. As a result, they were well suited to identifying what therapeutic content emerged (e.g., themes related to ED symptoms, trauma, and embodiment), but less able to explicate how these processes unfolded in time within the music-imagery experience, or how multiple therapeutic mechanisms interacted dynamically within individual sessions [54].

The use of case illustrations in the present study addresses this gap by enabling process-oriented, fine-grained examination of GIM as an experiential and relational intervention. Specifically, the case-based approach allows for: (a) tracing the unfolding of imagery in direct interaction with the music, (b) examining the interplay between symbolic, somatic, and affective processes as they emerge moment-to-moment, (c) highlighting the role of therapist attunement and intervention within the unfolding experience, and (d) illustrating how ED symptoms are enacted, represented, and potentially transformed within the context of a session.

### Case selection

A purposive sampling strategy was used to select four cases from the larger dataset (*N* = 8; 116 session transcripts) to illustrate the theoretical framework guiding this paper. Cases were selected based on the following criteria: (a) presence of imagery explicitly related to ED symptom function (e.g., restriction, bingeing, purging, or body-related distress), (b) clear engagement with trauma-related affective, or somatic material, (c) richness and coherence of imagery sequences that demonstrated symbolic or embodied processing, and (d) representation of variation in age, treatment level, and clinical presentation. To maintain analytic focus, excerpts were selected from sessions in which ED-relevant imagery was most salient and therapeutically developed.

### Participants and setting

Participants in the parent study included individuals engaged in ED treatment in a Midwest metropolitan area. Eligible individuals were invited to participate in the study if they were actively engaged in treatment (residential, intensive day or outpatient) and their treatment team determined GIM was an appropriate therapeutic approach given their engagement and response to treatment. Approval for the study was obtained through the Institutional Review Board (IRB) at the University of Minnesota. Informed consent for the study also included secondary data analyses, case study analysis and use of quotes. Exclusion criteria included: (a) non-English speaking, (b) medically unstable, and (c) GIM was contraindicated due to dissociative tendencies or dissociation. Flyers about the study were posted at the treatment programs and clients interested in participating needed to obtain approval from their primary therapist to discuss with the interdisciplinary treatment team and determine if GIM was an appropriate therapeutic intervention at this point in their treatment process.

After confirmation from the team, a research assistant met with participants to complete informed consent which also specified consent for the use of quotes from session transcripts and postlude reflections to be included in publications. Following the completion of the informed consent, the research assistant scheduled individuals for their initial session with the board-certified music therapist (MT-BC) facilitating the sessions. Participants maintained their regular treatment programming, while receiving GIM sessions. This included transitioning to different levels of care when appropriate and necessary. Eight clients indicated their desire to participate in the parent study, completed informed consent, and received a series of GIM sessions during their ED treatment.

## Materials

GIM sessions took place over a 12-month period while they were simultaneously engaged in various levels of ED treatment. The frequency of GIM sessions were determined based on individual therapeutic needs, pacing appropriate for each participant, and coordinated with their regular treatment programming. Overall, this resulted in GIM sessions occurring on a weekly or bi-weekly basis. Sessions were tailored to accommodate the 50-minute therapeutic hour, which required shortening some music programmes. Treatment fidelity was maintained by structuring sessions with the following components and tracking them on the session transcripts:10-12-minute check-in, 25-30-minute music and imagery experience, 10–12-minute postlude [50, 54]. All sessions were held in a private therapy office with a sofa and recliner, which allowed participants to choose if they wanted to sit reclined or lay down for the music and imagery sessions. Pillows and blankets were also available if participants preferred to use them for their comfort. The music utilised for the study was Music for the Imagination [74], music specifically developed for GIM and played from an Apple iPad Mini and through a Bose Bluetooth speaker.

### Analysis of case illustrations

The present analysis is theoretically driven and interpretive, integrating prior thematic findings with close, case-based examination of selected excerpts. A two-stage analytic process was employed: (1) identification of ED-relevant imagery segments within selected cases, and (2) interpretive mapping of these segments onto key constructs within the proposed trauma-informed, neuroaesthetics, and embodied framework. Analysis focused on how music-evoked imagery facilitated processes such as symbolic emotional exploration, somatic activation and integration, interoceptive awareness, affect regulation within safety, and meaning-making related to identity and attachment.

Attention was given to the dynamic interplay between music, imagery, therapist, intervention and client response, with emphasis on how ED symptoms were represented, experiences and transformed within the music and imagery process. To preserve contextual integrity, excerpts include client imagery descriptions, therapist prompts, and postlude reflections, enabling the examination of both in session processes and immediate integration.

## Results

Parent study participants received a total of 116 GIM sessions, which resulted in a range of between 11 and 17 GIM sessions over the course of the range of 12 to 40 weeks they were engaged in ED treatment. All 116 transcripts were analysed previously and the imagery themes that resulted from the thematic and intertextual analysis have been published elsewhere [49]. The thematic analysis explicated imagery related to ED symptoms and illustrated various ways they explored and worked through their symptom-based imagery. Across the 116 session transcripts nearly 30% included imagery related to ED symptoms.

Case illustrations with excerpts from four study participants are examined. During each GIM session, the therapist maintained a written transcript. Each transcript includes insights shared at the check-in, the music program, each piece of music utilised in the session, the relaxation experience and/or starting image introduced to initiate the imagery, the client’s description of the imagery, the therapist’s questions and supportive comments, and a summary of the reflection following the music and imagery experience.

Each case illustration includes background information related to the client’s diagnoses, ED history, previous treatment episodes, and information from the check-in. The piece(s) of music the client was listening to as their imagery experience unfolded is included. The introduction identifies the starting image introduced to the client as the music listening begins. The choice of the starting image was informed by the check-in and the client’s therapeutic needs. The music and imagery excerpts only include the portion of the GIM session where the imagery relates to the ED symptom. In the excerpts, clients’ descriptions of their experience are in normal text, questions and comments from the therapist are in italicised text and in parentheses and the clients’ actions are included in brackets. The postlude is an overview of reflections and insights immediately after each session.

### Case illustration1: emotion regulation

Participant 1 was a 28-year-old single woman diagnosed with AN binge-purge type, generalised anxiety disorder (GAD), obsessive-compulsive disorder (OCD), post-traumatic stress disorder (PTSD). She had been living with her ED for 13 years and had 9 previous ED treatment episodes. She arrived for her weekly session and as soon as she entered the therapist’s office she stated she needed to throw up. She sat down and I moved the waste basket next to her. As she sat on the couch, she appeared anxious with her left knee bouncing constantly. At this point in her therapeutic process, she had engaged in several GIM sessions and had been able to explore difficult experiences. Based on this previous work, I asked if she was willing to explore what was causing her to feel like she needed to throw up. She had 15 GIM sessions throughout the course of the study, this session excerpt is from session 12.

The music program selected for this session was entitled *Solace* [74] and comprised of six pieces of music. The music for this program is designed to support the imager in accessing and exploring deeper emotions. The music program was chosen to provide the musical structure, containment, and support for Participant 1 to safely enter and explore what was causing the urgency and feeling for the need to purge. The Swan of Tuonela is the second piece in the program, its slow tempos invite a slowing down, while the sustained and gently shifting textures create a sense of quietness and foster an inward focus and reflective state. The airy and sustained sound of the strings creates a feeling of being suspended in time and of quiet contemplation, fostering introspection.

Sibelius: Swan of Tuonela Introduction to session: Listening to what your body needs.

Music and Imagery experience excerpt: (*Notice*) My stomach is saying throw up. It wants to be empty. It hurts. (*What is there? )* Food. (*Anything else? )* Yes, but I don’t know what. I feel really full. There is a lot of sadness. (*Sadness… can you see it? )* Yes, it is dark blue, thick and heavy. It goes in and fills in all the spaces around the food. It is sticky and it’s stuck. (*How does it feel? )* Heavy. (*Where do you feel that?*) In my chest, shoulders, and stomach. My whole body feels tired. (*Let yourself take some slow breaths*). I need this thick substance to thin out so it can move. (*Ah*,* can you thin it out? )* It begins to thin out as I cry, and when I talk about it. Now it feels stuck again. (*Ah*,* remembering*,* crying and talking help to thin it out*). When it was thinner I could do those things and I felt safer then. (*Safer*). If I remember to connect with my feelings, and let the tears come, it thins out and I don’t feel full or stuck. (*Can you remember and let the tears come now?*) It’s hard. I feel the sadness that is there. [She begins to cry]. (*What’s happening now?*) As my tears fall, they thin out the thick, sticky goo. My tears wash it away. (*They wash it away*). Yes, my tears wash it all away. (*How does that feel?*) It hurts to feel the sadness and pain, and it is what I need to do to heal. (Your tears help you to heal). Yes, my tears help me heal.

Postlude: Following the music and imagery experience, she shared she did not feel she needed to throw up any longer. She verbalised she felt tired from all the crying, but she did not feel the fullness or pain in her body that the unresolved emotions had caused. She was able to reflect on how feeling and beginning to express these emotions that she had been avoiding and suppressing, allowed her to release them from her body.

### Case illustration 2: avoiding embodiment

Participant 2 was a 26-year-old single woman diagnosed with AN-restricting type, GAD, major depressive disorder and PTSD. She reported her ED symptoms began when she was 11 years old. She shared that as a very young child she had been the victim of ritualistic abuse at the hands of religious leaders. She reported having difficulty working through her trauma in previous verbal therapy and had come to believe that would not be able to find a way to work through this experience from her early childhood. She shared that she felt her body holding the trauma, but she did not know how to access it and work through it. She had a history of self-injurious behaviours and reported that cutting was her way of trying to remove the trauma from her body. She had been through 7 previous ED treatment episodes. She had 12 GIM sessions throughout the course of the study, the session excerpt is from session 10.

The music program selected for this session was, *Caring* [74] and comprised of six pieces of music. This music was chosen for the session as it helps to deepen an imagers consciousness, fosters a feeling of trust, and supports exploring aspects of childhood.

Haydn’s Cello Concert in C fosters a sense of intimacy and nurturance through the low, soothing and sustained tones of the cello. The light and transparent orchestral accompaniment supports the cello, creating a sense of connection and fostering a subtle emotional dialogue and mutual support. The steady rhythm and unhurried pulse support a calm introspection, while the gradual crescendos (louder) and decrescendos (softer) support emotional moderation, exploring and expressing feelings without being overwhelmed.

Haydn: Cello Concerto in C Introduction: Soft blanket giving you comfort.

Music and imagery experience excerpt: (*What do you notice?*) I feel the softness of the blanket on my skin. It feels so comforting and soothing. But I don’t feel good inside. (*What do you feel inside?*). An emptiness. (*Can you see it?*) As I look inside my body, I see that it is all charred. I feel the devastation and loss. (*How does it feel to see this?*). I feel sad and angry. I don’t know if this can be healed or if it can recover. I want to wash the charred ash away. (*Can you?*) I don’t know. I am stepping into a warm pool and letting the water flow over me and in through my skin. (*How does it feel?*) The water is warm and energizing. I feel the warmth on my skin and inside. I can see it is washing away the charred ash and revealing healthy flesh underneath. Underneath all that dark ash there is life. (*How does it feel to see that there is life there?*) I feel relieved, I didn’t think there was any life left there. I am sad I avoided this place and let my fear and sense of loss take away my hope recovering what I lost.

Postlude: When the music and imagery experience ended, she reflected on feeling surprised that this part of her could be restored. She assumed she would live the rest of her life, knowing what had happened to her caused this devastation and loss and that it could never be healed. She shared feeling a sense of empowerment in being able to discover a way to help this part of her heal and how her decision to step into the warm pool fostered this healing.

### Case illustration 3: self-protection

Participant 3 was a 57-year-old married woman diagnosed with BED, MDD, and GAD and reported using ED symptoms since her mid-20s. This was her fifth treatment episode. She described her childhood as difficult and lonely because her mother battled untreated schizophrenia. She shared that due to her mother’s fragile mental health state, she often felt unsafe. She frequently spent time alone and when she reached adolescence, she started sleeping with a knife under her pillow for fear her mother may attack her in the night. She shared memories of coming home from school and seeing her mother talking to the oven or to the sofa. This lack of safety in her relationship with her mother, also fostered feelings of being unloved. She recognized that the lack of attachment with her mother and feeling unloved made it difficult for her to trust that others loved her. She felt she did not have the ability to allow her heart to love and trust others. She had 16 GIM sessions throughout the course of the study, this excerpt is from session 11.

The music program chosen for this session was, *Positive Affect* [74] and comprised of five pieces of music. This program was chosen as the music and musical elements invite the imager into a more intimate reflection, to dwell and linger. Elgar’s Serenade for Strings introduces warm tones from the viola that foster an inward focus through its long and arching phrases. The subtle push and pull from the harmonic tension (dissonance) and release (resolution into harmony), mirrors emotional yearning and creates a feeling that there is something to explore. Further, the muted string orchestration fosters a sense of closeness and emotional warmth, creating a sense of safety and security, while the slow rhythmic tempo throughout supports a gradual forward movement.

Elgar: Serenade for Strings Introduction: Notice your heart.

Music and imagery experience excerpt: (*What do you notice?*) I am thinking I am sorry. I am sorry. I can feel my heart sink. (*How do you feel? )* It feels like when someone hurts you or does something mean to you. There is a lot of yuck in my heart. (*Can you see it?*) No, I feel it. (*Feel it?*) It’s like there are big rocks or weights in my heart, they make it heavy and unable to move and feel. (*Can you touch it?*) Yes. (*What do you notice?*) I grab one and it is not hard. It is like slime and it is sticky in my hand. (*How does it feel to touch it?*) Yuck! (*What do you want to do with it?*) I want to flick it off my hand. (*Can you?*) Some of it. It’s like silly putty and it sticks to the carpet. Yuck! Now it is everywhere. It’s dirty, dirty everywhere. There is a thousand times more in my heart. (*How are you feeling as you see it?*) Rage. (*Do you feel that in your body?*) Yes, in my legs, my head, and my stomach. I am so angry that my heart is full of this mess. I want it out. (*Can you get it out?*) I keep reaching in to grab another rock and it turns to slime. I pull out one after another and it oozes into my hands and drops to the floor. (*What do you notice?*) As I get more of them removed, I can begin to see space in my heart and my heart feels lighter. (*Space*) Space that I can decide what I want in my heart. I don’t want to carry that heaviness and hurt in my heart anymore. I need to decide what I want to carry. (*What do you want to carry?*) I want to carry love. I need to learn how to do that.

Postlude: During the reflection she shared that she noticed the weight she had been carrying (rocks in her heart). What began as heaviness and yuckiness in her heart was revealed as old hurts and pain that was overwhelming. The hurt turned into anger as she realized how much of it was still inside. Reaching in and pulling it out the rocks was difficult and uncomfortable, but with each piece she removed she felt space opening and a sense of lightness. She shared that creating that space reminded her that she has a choice about what I hold onto and what to let go of. She talked about not wanting her heart to be filled with this heaviness anymore but wanting to feel love. She recognized this was still something she was learning to do and acknowledged needing to tend to her needs and feeling empowered knowing that she can help her heart heal and that she can change.

### Case illustration 4: seeking validation

Participant 4 was a 54-year-old married woman with a 12-year history of AN-restricting type and diagnosed with MDD, GAD, and OCD. She reported longstanding struggles with perfectionism that emerged in early adolescence. She shared that her recurrent depressive episodes and chronic anxiety contributed to her restrictive eating patterns. This was her 13th ED treatment episode, and she shared that her family were quite frustrated with her due to the ongoing ED struggles and need for additional treatment. She shared that as a result she often feels lonely and alone in her struggles. She had 14 GIM sessions throughout the course of the study, the session excerpt is from session 10.

The music program selected for this session was *Relationships* [74], it was chosen as it supports exploring various interpersonal relationships, as well as one’s personal history and memories. In Chopin’s 1st Piano Concert, the piano is a lyrical voice fostering a sense of the personal and serves as an emotional driver. The soft and ornamented nature of the melody creates a feeling of hovering rather than moving forward. The orchestral accompaniment is sparse and transparent creating an effect of protected intimacy and providing a safe and private emotional space. Transitioning to Rachmaninoff’s 2nd Symphony, the music creates an emotionally immersive experience through its warm and long arching phrases. The harmonic lushness, modulations (changing tonal centre) and delayed resolutions stretch out and prolong tension, fostering a sense of aching and moving toward catharises. This engages the imagery into a sustained emotional experience, which can be explored and gradually move into release.

Chopin: 1st Piano Concerto in E minor & Rachmaninoff: 2nd Symphony (Adagio) Introduction: Doorway.

Music and imagery experience excerpt: (*What is happening?*) I opened the door and walked through it. I am standing in this space that is dark and cold. (*What do you notice?*) I see a light coming in from way up above me. As I look closer, I can see that I am at the bottom of a deep well. (*How do you feel?*) I feel cold and alone. I can’t see or find the doorway I came through, and it is so far up to the top. I sit down and cry. (*Let the music be with you here.*) No one will come to find me. I am just going to be left here. (*How are you feeling?*). I feel sad and so alone. (*Do you feel that in your body?*) Yes, I feel a heaviness in my heart and an emptiness in my gut. (*Allow yourself to breathe with the music*). [She takes several deep breaths]. (*What’s happening now?*) I hear voices in the distance. (*Voices*). They seem to be coming from way up at the top of the well. They are soft and faint but there are people there. (*Is there anything you want to do*?). I want them to know I am here. (*Can you tell them?*) I yell up that I am here. I am stuck at the bottom. They tell me they are here for me. They are waiting for me. It’s my family. (*Your family*).

{Music transitions to Rachmaninoff: 2nd Symphony} I didn’t think they cared, but they are here for me. (*Is there anything you want to do?*) I ask them to get me out. (*Can they?*) They say they can’t get me out, that I need to find my way out. I can’t climb to the top. I am stuck here. (*How does it feel to be stuck?*) I feel mad and sad. I can’t do this; I can’t get out of here on my own. (*What do you need?*) I need them, I need their help. (*Can you tell them?*) I tell them I can’t get out of here and I need their help. They tell me to climb toward the top and they will reach out their arms to pull me out. (*Can you do it?*) I don’t think so, but I have to try or I will be stuck here forever. I just start digging my fingers and toes into the soil of the wall to climb up. I lose my grip at times and slip. (*How does it feel?*) It is so slow and so hard to keep going. (*What do you notice?*) I see more of the light shining in as I climb up. (*How does it feel to see the light?*) It helps me to keep going and gives me hope. (Ah, hope). I am getting closer to the top even though I keep slipping. I can begin to see shapes of figures in the light. There are people gathered all waiting for me. (Waiting for you) Yes, they are waiting for me. They reach out their arms and help to pull me out. They tell me they have always been here waiting for me. (*How does it feel to here that?*) I feel so loved and cared for and I am sad that I did not know they have been there all this time.

Postlude: After the music and imagery experience, she shared how devastated and alone she felt in the well. She shared she often feels alone in her struggles with her ED and being at the bottom of the well mirrored how she often feels with her family. She discussed how surprised she was when she discovered in the experience that her family had always been there. She recognised that when she was stuck at the bottom of the well, she could not see them and assumed they were not there. She stated she was overjoyed to discover they were there and they welcomed me with open arms. She felt so loved, and realized she wanted them to rescue her, but she needed to be willing to get herself out of the hole she was in and climb toward them.

## Discussion

The case illustrations highlight the potential of GIM as a creative and embodied therapeutic approach that facilitates exploration of the underlying aetiology ED symptoms, enabling an individual to identify and externalise ED-related behaviours. Systemic examination of the clinical case material offers an in-depth analysis of these complex presentations and explicates the psychological, relational and neurobiological mechanisms that may maintain them. Within the four case excerpts, the functions and multifaceted roles of ED behaviours are explicated through the music-evoked imagery. Collectively, the cases demonstrate how an approach integrating creative, embodied, and trauma-informed perspectives supports a neuroaesthetics process through which previously inaccessible implicit material may become accessible to conscious awareness.

### Music-evoked imagery, emotional regulation and embodied affect

Difficulty with emotional regulation is a common feature of EDs, that can contribute to restriction, bingeing, purging, and compulsive behaviors [77, 78]. In the case illustrations, clients described intense affective states (sadness, rage, fear, emptiness, and grief) and how they experienced them somatically. The therapist supported music and imagery approach encouraged the clients to explore these emotions rather than avoid them, providing opportunities to practice engaging with their emotional landscape [49, 54, 79]. As the music and imagery experiences unfolded, they explored how their emotions were symbolically represented, enabling them to exert control over their engagement with them, and determine the distance from which they could safely tolerate the emotional intensity [53, 80]. This symbolic transformation of affect into imagery reflects a neuroaesthetics process whereby sensory, emotional, and cognitive inputs are integrated into metaphorical forms that can be more safely engaged with and regulated [79]. As a result, across the case illustrations GIM afforded clients opportunities to not only access, but also regulate, and transform affective experiences underlying their ED symptoms. These processes reflect the core therapeutic mechanism of GIM, particularly embodied emotion integration, interoceptive reconnection, and the develop of a safe and supportive affective space through which the client can gradually expand their window of tolerance for difficult emotional states [54, 80]. These findings align with previous research demonstrating GIM’s capacity to foster emotional regulation, insight, and resolution of unresolved intrapersonal conflicts across mental health and substance use disorder populations [47, 75]. These findings support research indicating that aesthetic and multimodal approaches can allow emotions to be symbolized, safely sensed, explored, and transformed within a contained therapeutic structure [49, 52, 53].

This is illustrated in Participant 1’s imagery of the thick, sticky substance that represented her sadness and which she experienced as physical fullness. This imagery represents the embodied nature of unresolved affect that drives her symptomatic behaviour. This experience of engaging with the sticky substance is indicative of interoceptive reconnection, as diffuse emotional distress becomes localised and perceptible within the body, allowing the client to identify, track, and engage with previously unexpressed internal states. The sustained low strings in the music and static harmonies evoke a sense of heaviness and emotional stuckness, mirroring a sense of fullness and sadness within the body. The mournful melody and gradual dynamic shifts support the expression of internalised sorrow. The music’s rhythmic and melodic return to stillness supports a sense of feeling and release. These musical elements do not merely mirror the client’s internal state but actively scaffold regulation by pacing emotional intensity, providing containment through the inherent predictability in the music, and support movement between activation and release. Through the music-supported imagery experience, she found that acknowledging and expressing these emotions relieved her somatic discomfort and reduced her urge to purge. This reflects the establishment of a safe and supported affective experience in which previously intolerable emotions could be experienced without the need for maladaptive regulation strategies. The process of emotional acceptance and experiencing and expressing her feelings, helped enable her to transform her unresolved emotions into a valuable insight [50, 54, 79].

The process observed in the case material are supported by neurobiological evidence demonstrating music’s capacity to engage multiple neural systems simultaneously, including affective, sensory, interoceptive, and autobiographical memory networks, which facilitate integrated processing of emotion, embodied experience, and meaning-making [71, 80, 81]. This may explain how participants were able to shift from undifferentiated somatic distress (e.g., fullness, heaviness) to differentiated symbolic imagery (e.g., thick, sticky substance), enabling more adaptive emotional processing. In this way, music-evoked imagery can serve as a bridge between somatic and emotional awareness, enhancing affect regulation and help in fostering an emerging coherent meaning of one’s internal experience, particularly for individuals whose emotions may be experienced as overwhelming [79–84]. Further, functional neuroimaging evidence indicates that music activates limbic and paralimbic regions and reward pathways, which can support emotional evaluation, interoceptive awareness, and retrieval of emotionally salient memories, linking affective experience with sensorimotor and embodied processing within a unified framework [71, 79]. This is particularly relevant in ED populations, where interoceptive deficits often contribute to difficulties distinguishing between emotional and psychological states such as hunger, fullness, and affective distress. Additionally, from a neuroaesthetics perspective, music’s simultaneous activation of sensory, emotional, and cognitive domains indicates music-evoked imagery as a therapeutic medium that can help to transform fragmented experiences into metaphorical material for exploration, fostering adaptive affect regulation to support awareness and development of insight [85, 86]. As seen in the case illustrations, the transformation of affect into tangible imagery enabled clients to externalise, engage, and manipulate their internal experience, facilitating both emotional regulation and cognitive insight. Overall, this suggests that GIM may help to facilitate affect regulation through the embodied, symbolic and neurobiological processes, enabling individuals to process overwhelming emotions within a structured, multisensory, and therapeutically contained environment [49, 55–57, 87].

### Exploring identity, attachment and relational capacity

Challenges related to identity, attachment, and self-worth can frequently underlie ED symptoms, especially in individuals that have experienced neglect, disrupted caregiving, or relational trauma [88, 89]. Individuals with EDs can experience their disorder is enmeshed with their sense of self and recovery may necessitate rebuilding one’s sense of self grounded in their personal values rather than ED behaviours [90]. This process can be best supported when therapeutic relationships tend to the whole person and empower their sense of agency outside the ED narrative, which may also be connected to attachment and relational capacity [91]. The structured nature of the music and imagery experience, relational attunement, and the reflexive pacing can foster a safe and secure therapeutic environment that fosters engagement [84, 86, 92].

This is evident in Participant 3’s imagery of the rocks in her heart, which illustrated a long-standing relational wound and unmet attachment needs that were somatically encoded, and resulted in her emotional guardedness. The melancholic harmonies in the strings of the music provide a gently sinking melody line that mirrors the initial feeling of heaviness in her heart. As the music unfolds, subtle shifts in the texture (instrumental sounds) and phrasing (moving from denser to lighter passages with more open tonality), reflects the process of confronting, pulling out and releasing the ‘sticky’ emotional residue. The serene and spacious (open) musical resolution supports a sense of emotional clearing. Through the GIM experience, she could change self-imposed boundaries, suspend limits, and explore a possible self [49, 93]. In this process, her anger emerged as a mobilizing affect, facilitating the release of internalized pain and creating space for new possibilities. This dynamic interplay offered her the opportunity to explore different ways of responding [93].

Her postlude reflection indicates that this experience fostered insight into choice, self-compassion, and an emerging capacity to feel love for herself and others. These outcomes align with findings from feasibility studies of GIM in eating disorder treatment, in which participants described enhanced self-compassion, greater awareness of relational patterns, and shifts in internal narratives following music and imagery experiences [54, 79]. Her symbolic reorganization through the music and imagery helps to highlight how it can support identity reconstruction by simultaneously engaging affective, cognitive, and embodied dimensions, allowing meaning-making that fosters integration of self-experience and relational capacity [54, 86].

### Addressing the complex profile of eating disorders

The high incidence of trauma and PTSD in individuals living with EDs contributes to a complex profile, impacting embodiment, fostering dissociation, and impairing interoception [83, 84]. Recent evidence indicates that early childhood trauma can hinder and disrupt embodied signal processing, reducing interoceptive trust, and is associated with difficulties in emotion regulation [93–95]. This can lead to a habitual distrust and disregard of interoceptive cues. To effectively address these complex and embodied issues, engagement in creative and multisensory therapeutic experiences can help to promote the coordination of internal (interoceptive) and external (exteroceptive and sensorimotor) sensory input, to provide a safe and structured opportunity for individuals to exert agency over how they interpret and respond [86, 87].

The case of Participant 2 illustrates how GIM provided a structured way to engage with traumatic material that she had not been able to access through verbal therapy. The warm, nurturing tones and lyrical phrasing of the cello create a sense of comfort. The light and fluid melody with balanced harmony evoke a sense of gentle movement to foster moving forward safely in the imagery. The gentle strength and brightness that builds in the melody supports her shift in feeling relief and a sense of hope. Her imagery of a charred interior reflected her early childhood trauma and loss, while stepping into warm water symbolized a reparative experience. This is consistent with GIM research and findings indicating that music-evoked imagery can foster emotional containment, reduce dissociative responses, and help to support trauma integration through symbolic and sensory processing [52, 79, 84]. Her experience also highlights the role of agency in trauma recovery. Rather than feeling overwhelmed by traumatic memories, within the music and imagery experience, she was able to engage in a way that helped foster healing. This sense of agency and empowerment is a key mechanism in both a trauma-informed care approach and ED recovery [46, 52–54] and is consistent with emerging GIM research emphasising the methods capacity to foster accessing internal resources and helping to develop self-efficacy [49, 50, 54, 59].

This evidence indicates that an individualised and depth-oriented approach may be warranted to address the complex and intertwined nature of therapeutic issues. Engagement in a creative and multisensory therapeutic approach can help to promote the coordination of internal and external sensory input, foster a sense of agency as the client exerts control over how they cognitively shape the therapeutic issue and determine how to interact with and respond to these stimuli [72]. This symbolic and metaphoric images of the individual’s inner experiences can allow them to access the material and bring it from subconscious awareness into consciousness [86].

Creative arts therapies have demonstrated efficacy in helping to reduce PTSD symptom severity, suggesting that non-verbal, multisensory engagement may help facilitate processing of traumatic material that is not easily accessed through verbal means [96]. These therapeutic approaches align with embodied trauma models that emphasize the important role of sensorimotor and somatic processing in trauma recovery [93]. Further, creative and embodied interventions can foster a sense of mastery and agency through intentionally generated sensory feedback, that helps strengthen an individual’s connection with their internal experiences and the external world, supporting their adaptive regulation [97, 98].

Further, creative and experiential therapeutic processes that integrate symbolic and metaphoric imagery help to provide access to subconscious material and foster the emergence of meaning [85, 98]. Imagery rescripting and similar techniques can enable individuals to revisit and modify traumatic mental images and experiences, which can facilitate new affective and cognitive interpretations of these experiences [99, 100]. This meaning-making process is central to supporting trauma integration and helping to restore a coherent sense of self, especially when trauma has disrupted bodily awareness and emotional groundedness [101, 102].

### Limitations

There are several limitations to be considered when interpreting these findings. First, the use of case illustrations limits generalizability, as GIM experiences are individualized, and informed by each client’s unique history, therapeutic needs, client-therapist relationship, and the music selections. Additionally, the individualized and flexible nature of the intervention, including variability in pacing and music selection, which can limit the ability to standardize the approach and replicate findings across participants and settings. Second, the case illustrations were drawn from a small sample of clients who consented to participate in a feasibility study and as a result may represent individuals who were receptive to the approach. This limited demographic and contextual diversity of the sample may further constrain the transferability of the findings. Third, the absence of a control or comparison condition limits the ability to attribute observed therapeutic processes or outcomes specifically to the GIM intervention, as opposed to nonspecific therapeutic factors such as attention, expectancy, or the therapeutic alliance, and the influence of prior or concurrent treatments was not systematically controlled for. Participants may have integrated and discussed what emerged in their GIM sessions into other therapeutic programming, which could have bolstered the impact. Fourth, case illustrations are based on qualitative session data and post-session reflections, drawing upon personal therapeutic experiences from a parent study which did not systematically assess ED symptoms, emotion regulation, and recovery trajectories [44]; the retrospective nature of case analysis and reliance on interpretive qualitative methods introduces the possibility of analytic subjectivity and session data may not fully capture the complexity of in-session processes, including nonverbal and embodied experiences. Fifth, case illustrations may be subject to selection and presentation bias, as cases that are more clinically compelling or coherent may be included. Lastly, the therapist’s dual role in session delivery and data analysis increases the risk of potential for bias and the lack of long-term follow-up limits the understanding of the durability of observed therapeutic shifts and their relationship to sustained recovery.

### Clinical practice implications

Considering the literature and the case illustrations, there are several practice recommendations for the use of GIM in ED treatment. GIM should be implemented within a trauma-informed framework, with careful attention to pacing, modulation of emotional intensity, and the client’s window of tolerance [46, 49, 55]. Many individuals with EDs have experienced complex trauma and may be vulnerable to emotional flooding or dissociation, as a result, ensuring they are resourced to manage emotions necessary prior to embarking on trauma work, and preparation and post-session integration are essential components to support the client’s therapeutic process [95, 96].

GIM may be indicated for clients who demonstrate difficulty accessing emotions verbally, experience somatic distress linked to ED symptoms, or present with disrupted interoception and embodiment. Introducing GIM after symptom stabilization and therapeutic alliance-building can enhance safety and effectiveness, especially for clients with long-standing ED history or recurrent treatment episodes [99, 103].

GIM therapists should closely attend to the symbolic content of imagery related to ED symptoms (e.g., fullness, emptiness, contamination, heaviness, or numbness), as these images offer clinically rich entry points for exploring underlying emotional, relational, and identity-based processes. Supporting clients in identifying moments of agency, choice, and internal resource building within the music and imagery experience can foster empowerment and development of new coping strategies they can extend beyond sessions [54–56]. Finally, the integration of GIM within multidisciplinary ED treatment settings is recommended in collaboration with the broader treatment team to support continuity of care, facilitate transfer of insights into daily life, and help clients translate embodied experiences into concrete behavioural and relational changes. Ongoing consultation and supervision are key for the GIM therapist to support their work with complex cases and therapeutic material, as well as to ensure ethical and effective practice.

## Future directions

These case illustrations support emerging evidence of GIM as a promising creative and embodied approach in ED treatment, particularly for individuals with complex trauma histories and clinical profiles. GIM’s integration of music and imagery to facilitate an embodied engagement and therapeutic attunement offers a unique means to address emotional dysregulation, impaired interoception, and fragmented self-experience that are key challenges in ED recovery [82, 86].

The literature on GIM in ED treatment remains limited, as result further reasearch is needed to further clarify clinical indications, optimal timing within treatment, and mechanisms of change. Mixed-methods studies and controlled trials would strengthen the empirical foundation and support systematic integration of GIM into multidisciplinary ED care. The cases help to explicate how ED symptoms can be explored and transformed through music-evoked imagery and highlight GIM’s potential as an embodied, aesthetic and relational approach that may support ED treatment and recovery.

## Data Availability

No datasets were generated or analysed during the current study.

## References

[1] Treasure J, Cardi V, Touyz S. Eating disorders. Lancet. 2024;403(10380):2142–54. 10.1016/S0140-6736(23)00426-3.

[2] Bongiorno D, Heaner S. Psychological and relational underpinnings of eating disorders. Intl J Eat Disord. 2025;58(2):123–38. 10.1002/eat.23456.

[3] Ortiz A, Peters A, Webber KT, Bulter R, Fitterman-Harris H, Levinson C. Eating disorder symptoms and corresponding evidence-based treatments: a narrative review. J Eat Disord 2026;14:45. 10.1186/s40337-025-01485-7 10.1186/s40337-025-01485-7 PMC12882398 41514362

[4] Gajperia P, Liang H, Smith J. Comorbidity patterns in eating disorder populations: A systematic review. J Eat Disord. 2024;12:Article 67. 10.1186/s40337-024-0076-3

[5] American Psychiatric Association. Diagnostic and statistical manual of mental disorders: DSM-5-TR. 5th ed. American Psychiatric Association; 2022.

[6] Smink FRE, van Hoeken D, Hoek HW. Epidemiology of eating disorders: Incidence, prevalence and mortality rates. Curr Psychiatry Rep. 2012;14(4):406–14. 10.1007/s11920-012-0282-y. 22644309 10.1007/s11920-012-0282-y PMC3409365

[7] Solmi M, Radua J, Olivola M, Croce E, Soardo L, de Pablo G, et al. Age at onset of mental disorders worldwide: large-scale meta-analysis of 192 epidemiological studies. Mol Psychiatry. 2022;27:281–95. 10.1038/s41380-021-01161-7. 34079068 10.1038/s41380-021-01161-7 PMC8960395

[8] Gollo Bertollo A, Ferreira Puntel C, Bastos Rievers HF, Serpa Brunhara EG, Ignácio ZM. Neurobiological and psychosocial mechanisms linking early life stress to pathogenesis of eating disorders. Neuroscience. 2026;296:128–142. 10.1016/j.neuroscience.2026.01.008 41525812

[9] Stover PJ, Field MS, Andermann ML, Bailey RL, Batterham RL, Cauffman E, et al. Neurobiology of eating behavior, nutrition, and health. J Intern Med. 2023;294:582–604. 10.1111/joim.13699. 37424220 10.1111/joim.13699 PMC13746983

[10] Abdoli M, Schiechtl E, Rosato MS, Mangweth-Matzek B, Cotrufo P, Hüfner K. Body image, self-esteem, emotion regulation, and eating disorders in adults: a systematic review. Neuropsychiatry. 2025;39(3):118–32. 10.1007/s40211-025-00544-4 PMC12397123 40830328

[11] Rantala MJ, Luoto S, Krama T, Krams I. Eating disorders: an evolutionary psychoneuroimmunological approach. Front Psychol. 2019;10:2200. 31749720 10.3389/fpsyg.2019.02200 PMC6842941

[12] Weems D. Exploring the interplay of biological psychological and social factors in eating disorders a comprehensive review. J Child Adolesc Behav. 2023;11(9):556.

[13] World Health Organization (WHO). Global health estimates: Leading causes of DALYs. 2024. https://www.who.int/data/gho/data/themes/ mortality-and-global-health-estimates/global-health-estimates-leading-causes-of- dalys. Accessed 25 Jan 2026.

[14] Umbrella Review. Global prevalence and trends in eating disorders 2000–2024: a systematic review. World Psychiatry. 2025;24(1):65–83. 10.1002/wps.2135.

[15] Santomauro D, Melen S, Mitchison D, Vos T, Whitford H, Ferrari A. The hidden burden of eating disorders: an extension of estimates from the global burden of disease study 2019. Lancet. 2021;8:320–8. 10.1016/S2215-0366(21)00040-7. 10.1016/S2215-0366(21)00040-7 PMC7973414 33675688

[16] Martin-Wagar C, Boswell R, Bennett B, Perelman H, Forrest L. Psychological and eating disorder symptoms as predictors of starting eating disorder treatment. Int J Eat Disord. 2021;54:1500–8. 10.1002/eat.23538. 33959999 10.1002/eat.23538

[17] Convertino M, Mendoza R. Trauma-informed approaches in eating disorder treatment. Eur Eat Disord Rev. 2024;32(1):45–63. 10.1002/erv.3000.

[18] Taquet M, Geddes JR, Sierra L, Harrison PJ. Incidence and outcomes of eating disorders during the COVID-19 pandemic. Br J Psychiatry. 2021;220(5):1–3. 10.1192/bjp.2021.105. 35048812 10.1192/bjp.2021.105 PMC7612698

[19] Hambleton A, Pepin G, Le A, Maloney D, Touyz S, Maguire S, National Eating Disorder Research Consortium. Psychiatric and medical comorbidities of eating disorders: findings from a rapid review of the literature. J Eat Disord 2024 Sept 5:10(1):132. 10.1186/s40337-022-00654-2. 10.1186/s40337-022-00654-2 PMC9442924 36064606

[20] Udo T, Grilo CM. Prevalence and correlates of DSM-5 eating disorders in a nationally representative sample of United States adults’, JAMA Psychiatry. 2019, 76(7), 715–725. 10.1001/jamapsychiatry.2019.0253

[21] Treasure J, Duarte TA, Schmidt U. Eating disorders. Lancet. 2020;395(10227):99–911. 10.1016/S0140-6736(20)30059-3. 10.1016/S0140-6736(20)30059-3 32171414

[22] Hudson JI, Hiripi E, Pope HG, Kessler RC. (2007) ‘The prevalence and correlates of eating disorders in the National Comorbidity Survey Replication’, Biol Psychiatry. 2007 Feb 1;61(3):348 – 58. 10.1016/j.biopsych.2006.03.040 PMC1892232 16815322

[23] Keel KP, Holland LA. Eating disorders. In Gotlib IH, Hammen CL, editors The Oxford Handbook of Depression and Comorbidity.2014. Oxford University Press, pp. 166–85. 10.1016/j.biopsych.2006.03.040

[24] Martinussen M, Friborg O, Schmierer P, Kaiser S, Øvergård KT, Rosenvinge JH. The comorbidity of personality disorders in eating disorders: a meta-analysis’, Eating and Weight Disorders – Studies on Anorexia. Bulimia Obes. 2017;22(2):201–9. 10.1007/s40519-016-0276-1. 10.1007/s40519-016-0345-x 27995489

[25] McGrath JJ, Lim C, Plana-Ripoll O, Holtz Y, Agerbo E, Momen N, et al. Comorbidity within mental disorders: a comprehensive analysis based on 145,990 survey respondents from 27 countries. Epidemiol Psychiatr Sci. 2020;29:e153. 32782057 10.1017/S2045796020000633 PMC7443806

[26] Kinnear A, Siegel JA, Masson PC, Bodell LP. Functions of disordered eating behaviors: a qualitative analysis of the lived experience and clinician perspectives. J Eat Disord. 2023;11:141. 10.1186/s40337-023-00854-4. 37605248 10.1186/s40337-023-00854-4 PMC10440936

[27] Brewerton TD. (2007). Eating disorders, trauma, and comorbidity: Focus on PTSD. Eat Disord. 2007 Jul-Sept; 15(4): 285–304. 10.1080/10640260701454311 10.1080/10640260701454311 17710567

[28] Velkoff E, Perkins N, Dodd D, Brown T, Kaye W, Wierenga C. Elevated interoceptive deficits in individuals with eating disorders and self-injurious thoughts and behaviors: A replication and extension. Suicide Life Threat Behav. 2024;54(1):129–37. 38009622 10.1111/sltb.13024

[29] Haynos AF, Fruzzetti AE. Anorexia nervosa as a disorder of emotion dysregulation: Evidence and treatment implications. Clin Psychol Sci Pract. 2011;18(3):183–202. 10.1111/j.1468-2850.2011.01250.x.

[30] Skårderud F. Shame and pride in anorexia nervosa: A qualitative descriptive study. Eur Eat Disord Rev. 2007;15(2), 81–97. 10.1002/erv.774. 10.1002/erv.774 17676677

[31] Molendijk ML, Hoek HW, Brewerton TD, Elzinga BM. Childhood maltreatment and eating disorder pathology: A systematic review and dose–response meta- analysis. Psychol Med. 2017;47(8):14021416. 10.1017/S0033291716003561 28100288

[32] Trottier K, MacDonald DE. Update on psychological trauma, other severe adverse experiences and eating disorders: State of the research and future research directions. Curr Psychiatry Reps. 2017; 19(8): 45. 10.1007/s11920-017-0806-6 28624866

[33] Kelly AC, Carter JC. Epub. Self-compassion training for binge eating disorder: a pilot randomized controlled trial. Psychol Psychother. 201588(3), 285–303. 10.1111/papt.12044. 10.1111/papt.12044 25330466

[34] Khalsa SS, Portnoff LC, McCurdy-McKinnon D, Feusner JD. What happens after starvation: a review of interoceptive processing in eating disorders. J Eat Disord. 2017;5:20. 28630708 10.1186/s40337-017-0145-3 PMC5470198

[35] Cogodi E, Ranieri J, Martelli A, Di Giacomo D. Emotional dysregulation in anorexia nervosa: scoping review of psychological treatments. Healthc. 2024;11(14):1388. 10.3390/healthcare12141388 PMC11276072 39057531

[36] Trompeter N, Bussey K, Forbes MK, Mitchison D. Emotion dysregulation within the CBT-E model of eating disorders: a narrative review. Cogn Therapy Res. 2021;45:1021–36.

[37] Zhou R, Zhang L, Liu Z, Cao B. Emotion regulation difficulties and disordered eating in adolescents and young adults: a meta-analysis. J Eat Disord. 2025;13:25. 39940059 10.1186/s40337-025-01197-y PMC11823190

[38] Selby EA, Bodell LP, Haynos AF. Positive emotion dysregulation in eating disorders and dysregulated eating behaviors. Front Psychol. 2024;15:1437889. 10.3389/fpsyg.2024.1437889 PMC11233818 38988386

[39] Spix M, Martijn C, Jansen A. Don’t check after a snack: body checking increases eating-related threat perception. Appetite. 2026;216:108307. 40967274 10.1016/j.appet.2025.108307

[40] Lian Y, Liu S, Zhang D, Qiao D, Zhang Z, Mi G, et al. Emotion dysregulation and eating disorder symptoms: a network analysis in college students with subclinical eating disorders. J Eat Disord. 2025;13(1):161. 40731292 10.1186/s40337-025-01325-8 PMC12309016

[41] Zhang J, Cui S, Zickgraf HF, Barnhart WR, Xu Y, Wang Z et al. A longitudinal network analysis of emotion regulation, interpersonal problems, and eating disorder psychopathology in Chinese adolescents. Int J Eat Disord. 2024;57(12):2415–2426. 10.1002/eat.24292 39364628

[42] Norton B, Sheen J, Burns L, Enticott PG, Fuller-Tyszkiewicz M, Kirkovski M. Overlap of eating disorders and neurodivergence: the role of inhibitory control. BMC Psychiatry 2024;24(1): 454. 10.1186/s12888-024-05837-6. PMID: 38890597; PMCID: PMC11186180. 10.1186/s12888-024-05837-6 PMC11186180 38890597

[43] Cobbaert L, Hay P, Mitchell PB, Roza SJ, Perkes I. Sensory processing across eating disorders: a systematic review and meta-analysis. Int J Eat Disord. 2024; 57(7):1465–1488. doi: 10.1002/eat.24184. 10.1002/eat.24424. 10.1002/eat.24184 38511825

[44] Kambanis PE, Mancuso CJ, Becker KR, Eddy KT, Thomas JJ, De Young KP. Course of avoidant/restrictive food intake disorder: emergence of overvaluation of shape/weight. J Eat Disord. 2024;12(1):54. 10.1186/s40337-024-01001-3. 38702736 10.1186/s40337-024-01001-3 PMC11067077

[45] Jewell T, Apostolidou E, Sadikovic K, Tahta Wraith K, Liston S, Simic M, et al. Attachment in individuals with eating disorders compared to community controls: a systematic review and meta-analysis. Int J Eat Disord. 2023;56(5):888–908. 10.1002/eat.23922. 36916409 10.1002/eat.23922

[46] Brewerton TD, Alexander J, Schaefer J. Trauma-informed care and practice for eating disorders: personal and professional perspectives of lived experiences. Eat Weight Disord. 2019;24(2):329–38. 30565188 10.1007/s40519-018-0628-5

[47] Heiderscheit A, Murphy K. Trauma-informed care in music therapy: principles, guidelines and a clinical case Illustration. Music Ther Perspect. 2021;39:2, 142–151.10.1093/mtp/miab011

[48] Grocke D, editor. Guided Imagery and Music (GIM): The Bonny Method and Beyond. 2nd ed. Barcelona; 2019.

[49] Bonny H. The story of GIM: The beginnings of the Bonny method of guided imagery and music. Barcelona; 1995.

[50] Heiderscheit A. Feasibility of the Bonny Method of Guided Imagery and Music (GIM) in eating disorder treatment: clients perceived benefits and challenges. Arts Psychother. 2023;86:102086. 10.1016/j.aip.2023.102086.

[51] Heiderscheit A, Short A, Trondalen G, Young L. An integration of physical and psychological health through the Hero’s Journey in Guided Imagery and Music: A cross-case analysis. The Arts in Psychotherapy. 2025;96:102348. 10.1016/j.aip.2025.102348

[52] Bruscia K. Addressing the risks of GIM. In: Bruscia K, editor. Notes on the practice of guided imagery and music. Barcelona; 2015. pp. 95–101.

[53] Heiderscheit A. GIM in the Therapeutic Hour and case illustration of an adult client in eating disorder treatment. In: Grocke D, Moe T, editors. Guided Imagery and Music (GIM) and Imagery Methods for Individual and Group Therapy. Jessica Kingsley; 2015. pp. 99–107.

[54] Heiderscheit A. Thematic and intertextual analysis from a feasibility study of the Bonny Method of Guided Imagery and Music with clients in eating disorder treatment. Front Psychol. 2024;15:1456033. 10.3389/fpsyg.2024.1456033. 39545138 10.3389/fpsyg.2024.1456033 PMC11560787

[55] Heiderscheit A. The effects of the Bonny Method of Guided Imagery and Music (GIM) on interpersonal problems, sense of coherence, and salivary immunoglobulin A of adults in chemical dependency treatment. Music Med. 2017;9(1):24–36.

[56] Heiderscheit A. Analysis of the type of imagery and imagery themes from Bonny Method of Guided Imagery and Music sessions with adults in chemical dependency treatment. Arts Psychother. 2022;80;101933.

[57] Moe T, Roesen A, Raben H. Restitutional factors in group music therapy with psychiatric patients based on a modification of Guided Imagery and Music (GIM). Nordic J Music. 2000;9(2):36–50. 10.1080/08098130009478000.

[58] Moe T. Group guided imagery and music therapy for inpatients with substance abuse disorder. J Association Music Imag. 2012;13:77–98.

[59] Borling J. (1992). Perspectives on growth with a victim of Abuse. A Guided Imagery and Music (GIM) case study. Journal of the Association of Music & Imagery. 1992. 1; 85–98.

[60] Clements-Cortes A, Breaking. free. Healing physical, verbal and sexual abuse through the Bonny Method of Guided Imagery and Music. Journal of the Association of Music and Imagery. 2014, 14: 9–60.

[61] Blake RL. Vietnam veterans with posttraumatic stress disorder: Findings from a music and imagery project. J Association Music Imag. 1994;3:5–18.

[62] Ventre M. Healing the wounds of childhood abuse: A Guided Imagery and Music case study. Music Therapy Perspect. 1994;12(2):98–103. 10.1093/mtp/12.2.98.

[63] Lee J. Using Music Breathing to support emotion regulation for patients in inpatient eating disorders unit. J Association Music Imag. 2020;20:1–22.

[64] Khalsa SS, Craske MG, Li W, Vangala S, Strober M, Feusner JD. Altered interoceptive awareness in anorexia nervosa: Effects of meal anticipation, consumption and bodily arousal. Int J Eat Disord. 2015;48; 7:889 – 97. 10.1002/eat.22387. 10.1002/eat.22387 PMC4898968 25712775

[65] Price CJ, Hooven C. Interoceptive Awareness Skills for Emotion Regulation: Theory and Approach of Mindful Awareness in Body-Oriented Therapy (MABT). Front Psychol. 2018;9:798. 10.3389/fpsyg.2018.00798. 29892247 10.3389/fpsyg.2018.00798 PMC5985305

[66] SAMHSA. Trauma-informed care in behavioral health services (TIP 57). Substance Abuse and Mental Health Services Administration. 2014. https://library.samhsa.gov/product/tip-57-trauma-informed-care-behavioral-health-services/sma14-4816. 24901203

[67] Van der Kolk BA. The body keeps the score: Brain, mind, and body in the healing of trauma. Viking. 2014.

[68] Bonny HL. Music and consciousness: The evolution of guided imagery and music (GIM). Barcelona Publishers. 2002.

[69] Grocke D, Wigram T. Receptive methods in music therapy: Techniques and clinical applications. Jessica Kingsley. 2007.

[70] Blood AJ, Zatorre RJ. Intensely pleasurable responses to music correlate with activity in brain regions implicated in reward and emotion. Proc Natl Acad Sci. 2001;98(20):11818–23. 11573015 10.1073/pnas.191355898 PMC58814

[71] Luo YJ, Yu Q, Wu S, Luo YJ. Distinct neural bases of visual art- and music-induced aesthetic experiences. NeuroImage. 2025;305:120962. 10.1016/j.neuroimage.2024.120962. 39638082 10.1016/j.neuroimage.2024.120962

[72] Koelsch S. Music-evoked emotions: Principles, brain correlates, and implications for therapy. Ann N Y Acad of Sci. 2015; 1337:193–201.10.1111/nyas.12684. 10.1111/nyas.12684 25773635

[73] Ogden P, Fisher J. Sensorimotor psychotherapy: Interventions for trauma and attachment. Norton. 2015.

[74] Bruscia K. Music for the Imagination. Barcelona Publishers. 1998.

[75] Bruscia KE, Grocke DE. Guided imagery and music: The Bonny method and beyond. Barcelona; 2002.

[76] McKinney C, Honig T. (2017). Health outcomes of a series of Bonny Method of Guided Imagery and Music sessions: A systematic review. Journal of Music Therapy. 2017, 54;1: 1–34. 10.1093/jmt/thw016 10.1093/jmt/thw016 27941132

[77] Lavender JM, Wonderlich SA, Engel SG, Gordon KH, Kaye WH, Mitchell JE. Dimensions of emotion dysregulation in anorexia nervosa and bulimia nervosa: A conceptual review of the empirical literature. Clin Psychol Rev. 2015; 40:111 – 22. 10.1016/j.cpr.2015.05.010. 10.1016/j.cpr.2015.05.010 PMC4537813 26112760

[78] Mallorquí-Bagué N, Vintró-Alcaraz C, Sánchez I, Riesco N, Agüera Z, Granero R, et al. Emotion Regulation as a Transdiagnostic Feature Among Eating Disorders: Cross-sectional and Longitudinal Approach. Eur Eat Disord Rev. 2018;26(1):53–61. 10.1002/erv.2570. 29168283 10.1002/erv.2570

[79] David S. Emotional Agility: Get Unstuck, Embrace Change, and Thrive in Work and Life. Avery. 2016.

[80] Vaisvaser S, King J, Orkibi H, Aleem H. Neurodynamics of relational aesthetic engagement in creative arts therapies. Rev Gen Psychol. 2024;28(3):203–18. 10.1177/10892680241260840.

[81] Ren Y, Mehdizadeh SK, Leslie G, Brown T. Affective music during episodic memory recollection modulates subsequent false emotional memory traces: an fMRI study. Cogn Affect Behav Neurosci. 2024;24(5):912–30. 38955872 10.3758/s13415-024-01200-0

[82] Putkinen V, Nazari-Farsani S, Seppälä K, Karjalainen T, Sun L, Karlsson HK et al. Decoding Music-Evoked Emotions in the Auditory and Motor Cortex. Cereb Cortex. 2021;31(5):2549–2560. 10.1093/cercor/bhaa373. 10.1093/cercor/bhaa373 33367590

[83] Kennett YN, Humphries S, Chatterjee A. (2023). A thirst for knowledge: grounding curiosity, creativity, and aesthetics in memory and reward neural systems. Creat. Res. J. 2023, 35; 412–426. 10.1080/10400419.2023.2165748

[84] Salvato G, Richter F, Sedeño L, Bottini G, Paulesu E. Building the bodily self-awareness: evidence for the convergence between interoceptive and exteroceptive information in a multilevel kernel density analysis study. Hum Brain Mapp. 2019;41:401–18. 10.1002/hbm.24810. 31609042 10.1002/hbm.24810 PMC7268061

[85] Beck BD, Messel C, Trondalen G. Music therapy and trauma: An overview of approaches and evidence. Nordic J Music Therapy 2018, 27;5:369–87. 10.1080/08098131.2018.1479006

[86] Vaisvaser S. The embodied-enactive-interactive brain: bridging neuroscience and creative arts therapies. Front Psychol. 2021;12:634079. 10.3389/FPSYG.2021.634079. 33995190 10.3389/fpsyg.2021.634079 PMC8121022

[87] Maack C, Nolan P. The effects of GIM on PTSD symptoms. J Music Ther. 1999;36:4: 249–69. 10.1093/jmt/36.4.249.

[88] Day S, Hay P, Tannous WK, Fatt SJ, Mitchison D. A Systematic Review of the Effect of PTSD and Trauma on Treatment Outcomes for Eating Disorders. Trauma Violence Abuse. 2024;25:2:947–64. 37125723 10.1177/15248380231167399 PMC10913314

[89] Keski-Rahkonen A. Dissociative and traumatic experiences in people with eating disorders. Curr Opin Psychiatry. 2025;38(6):427–433. doi: 10.1097/YCO.0000000000001032. 10.1097/YCO.0000000000001032 40778697

[90] Conti JE, Joyce C, Hay P, Meade T. (2020). Finding my own identity: A qualitative metasynthesis of adult anorexia nervosa treatment experiences. BMC Psychology, 2020; *8*, 110. 10.1186/s40359-020-00476-4 10.1186/s40359-020-00476-4 PMC7583290 33092638

[91] Jewell T, Apostolidou E, Sadikovic K, Tahta-Wraith K, Liston S, Simic M et al. (2023). Attachment in individuals with eating disorders compared to community controls: A systematic review and meta-analysis. *Inter J Eat Disord*, 2023; *56*:5, 888–908. 10.1002/eat.23922 10.1002/eat.23922 36916409

[92] Grocke DE, Moe T, editors. Guided imagery and music (GIM) and music imagery methods for individuals and groups. Jessica Kingsley Publishers. 2015.

[93] Shankar M. The other side of change: Who we become when life makes other plans. Penguin Random House; 2026.

[94] Schmitz M, Back SN, Seitz KI, Harbrecht NK, Streckert L, Schulz A, et al. The impact of traumatic childhood experiences on interoception: disregarding one’s own body. Borderline Personal Disord Emot Dysregul. 2023;10(1):5. 36788573 10.1186/s40479-023-00212-5 PMC9930318

[95] Romano KA, Peterson CB, Anderson LM, Richson BN, Dougherty E, Heron KE. Affect, interoception, and disordered and intuitive eating behaviors: Examining momentary mediational associations among women with eating disorder pathology. Appetite. 2026;217:108342. 41077072 10.1016/j.appet.2025.108342 PMC12554365

[96] Piran N. Journeys of embodiment at the intersection of body and culture. The developmental theory of embodiment. Elsevier Academic; 2017.

[97] Heiderscheit AGIM. Deprivation and its Contribution to Pain in Eating Disorders. In: Mondanaro J, Sara G, editors. Music and Medicine: Integrative Models in the Treatment of Pain. Satchnote; 2013. pp. 147–71.

[98] Trondalen G, Bonde LO. Music Therapy: Models and Interventions. In: MacDonald R, Kreutz G, Mitchell L, editors. Music, Health and Wellbeing. Oxford University Press; 2012. pp. 40–61.

[99] Heiderscheit A. The Bonny Method of Guided Imagery and Music (GIM) in eating disorder treatment. In Heiderscheit, A, editor. Creative Arts Therapies in Eating Disorder Treatment, 120–141. Jessica Kingsley Publishers. 2015.

[100] Visco-Comandini F, Papa C, Uvelli A, Mancini F, Pugliese E. (2025). Rewriting trauma: A systematic review of treatment effects of imagery rescripting for PTSD and complex PTSD. European Journal of Trauma and Dissociation. 2025; *9*:4, 100609. 10.1016/j.ejtd.2025.100609

[101] Nader P, Ghadieh HE, Abbas N, Nahas N. Attachment and emotional eating: a scoping review uncovering relational roots to inform preventive healthcare. Healthc (Basel). 2025;13(23):3170. 10.3390/healthcare13233170 PMC12692234 41373387

[102] Musolino CM, Warin M, Gilchrist P. Embodiment as a Paradigm for Understanding and Treating SE-AN: Locating the Self in Culture. Front Psychiatry. 2020;11:534. 32595537 10.3389/fpsyt.2020.00534 PMC7304294

[103] Heiderscheit A, Gawronski J, Bloska J, Milton T, Ragnhildstveit A, Neufeld S. Music-based interventions in eating disorder treatment: A scoping review. Front Psychiatry. 2025;16(1660696). 10.3389/fpsyt.2025.1660696. 10.3389/fpsyt.2025.1660696 PMC12504190 41069947

